# Gestational cardiovascular health and adverse pregnancy outcomes: a prospective cohort study in China

**DOI:** 10.1080/07853890.2026.2703887

**Published:** 2026-07-26

**Authors:** Qi Wu, Hao Ma, Yihui Wu, Shuqi Zhu, Jiayue Tang, Mengjia Hu, Yibo Tang, Luyao Hu, Lixia Zhang, Xiaolin Xu, Danqing Chen, Lu Qi, Zhaoxia Liang

**Affiliations:** ^a^ Obstetrical Department, Women’s Hospital, School of Medicine, Zhejiang University, Hangzhou, China; ^b^ Department of Epidemiology, School of Public Health and Tropical Medicine, Tulane University, New Orleans, LA, USA; ^c^ Zhejiang Key Laboratory of Maternal and Infant Health, Hangzhou, China; ^d^ School of Public Health, Zhejiang University, Hangzhou, China

**Keywords:** Cardiovascular health, adverse outcomes, Chinese women

## Abstract

**Background:**

Pregnancy is regarded as a crucial window period for the assessment and management of cardiovascular health (CVH). This study investigated the relationship between the gestational CVH metrics of Chinese women and adverse pregnancy outcomes.

**Patients and methods:**

Six separate CVH metrics (smoking status, body mass index, blood pressure, total cholesterol, fasting blood glucose, and sleep health) were measured at 24–28 gestational weeks and categorized as ideal (2 points), intermediate (1 point), or poor (0 point) to generate a gestational CVH score. Total gestational CVH was categorized as high (all ideal metrics), moderate (no poor metrics), or low (one or more poor metric). Associations between gestational CVH and adverse pregnancy outcomes were evaluated using Poisson regression.

**Results:**

Among the 2,775 pregnant women included, 36.07%, 36.11%, and 27.82% had high, moderate, and low CVH, respectively. Each one-point increase in the CVH score was significantly associated with 7% and 4% lower risk of adverse maternal and neonatal outcomes (relative risk [RR], 0.93 and 0.96; 95% confidence interval [CI], 0.90–0.95 and 0.93–0.98). Compared with low CVH, high CVH was associated with 21% and 12% lower risk of adverse maternal and neonatal outcomes (RR, 0.79 and 0.88; 95% CI, 0.72–0.87 and 0.80–0.96). Individual CVH metrics also showed associations with adverse pregnancy outcomes, in which ideal body mass index, fasting blood glucose and sleep health were particularly important.

**Conclusions:**

High gestational CVH was significantly associated with a considerably lower risk of adverse pregnancy outcomes, but approximately two-thirds of pregnant Chinese women do not achieve a high level of CVH.

## Introduction

Pregnancy is considered a critical period for cardiovascular health (CVH) assessment and management. A large body of evidence has shown that poor gestational CVH is associated with higher risks of hypertension, diabetes, and other cardiovascular and metabolic complications in the future [1]. A presidential advisory from the American Heart Association (AHA) and the American College of Obstetricians and Gynecologists stated that pregnancy was a physiological ‘stress test’ of potential risk for cardiovascular disease [2]. Maternal cardiometabolic risk factors (e.g. obesity, hyperglycemia, hyperlipidemia, and hypertension) may first appear during pregnancy and have persistent and specific effects on maternal and fetal health [3]. While it is possible that < 10% of pregnant women in the United States have a high level of CVH [4], the levels of CVH in pregnant women in other countries are poorly understood.

In accordance with AHA recommendations made in 2010 and revised in 2022, population CVH has been comprehensively evaluated using multiple CVH metrics including diet, physical activity, smoking status, sleep health (new metric), body mass index (BMI), blood lipids, blood glucose, and blood pressure [5,6]. In February 2023, an updated statement from the AHA noted that pre-pregnancy CVH was closely correlated with the risks of adverse outcomes and long-term disease in pregnant and postpartum women and their offspring. This scientific statement highlighted that the preconception period was a critical window period for optimizing CVH, providing more opportunities for intervention to improve pregnancy outcomes and health across the life course [7]. However, we cannot ignore the impact of gestational CVH either. Because of the dramatic hormonal and metabolic changes that occur [8], maternal CVH during pregnancy may also change compared with that before pregnancy, which may be more strongly and directly associated with pregnancy outcomes. At present, only a few studies have evaluated the CVH level of pregnant women, but studies on its association with pregnancy outcomes are still lacking. Furthermore, although the various studies used the term ‘CVH’, the scoring criteria were actually not consistent, especially for pregnant women. Amanda M. et al. [4] adopted a scoring criteria similar to that used for non-pregnant individuals, while the HAPO study relied more on its own research data as the classification standard [3]. This also led to heterogeneity in the research results.

Our study describes gestational CVH metrics in Chinese women and investigated the relationships between these metrics and adverse pregnancy outcomes. CVH was evaluated using a combination of six gestational metrics: smoking status, BMI, blood pressure, total cholesterol, fasting blood glucose, and sleep health.

## Patients and methods

### Study design and population

The data presented in this study are from an ongoing prospective cohort study: Gestational Weight Gain Criteria for Pregnant Women with Gestational Diabetes Mellitus in China (NCT 04744714). In total, 2,775 pregnant women who attended antenatal visits and gave birth in Women’s Hospital School of Medicine Zhejiang University from February 2021 to April 2023 were included in the study. The inclusion criteria were women with a single pregnancy who had completed a gestational questionnaire, with available clinical information at 24–28 gestational weeks and delivery medical records. Women with any severe disease prior to pregnancy that could threaten the life of the mother or fetus were excluded. The study adhered to the Declaration of Helsinki and was approved by the Ethics Committee of Women’s Hospital School of Medicine Zhejiang University (IRB-20200273-R). Written informed consent was obtained from all participants.

Gestational CVH metrics were assessed using a gestational questionnaire and clinical information obtained at 24–28 gestational weeks to determine smoking status, BMI, blood pressure, total cholesterol, fasting blood glucose, and sleep health. Similar to the Hyperglycemia and Adverse Pregnancy Outcome (HAPO) study [3,9], diet and physical-activity metrics were not included in our study because the relevant data were not collected. Maternal age, gravidity, parity, occupation, income, and education data, which were the common clinical factors associated with pregnancy outcomes [10–14], were also gathered.

The primary objective of this study was to combine the above six metrics to assess the CVH of pregnant women and to explore their associations with adverse pregnancy outcomes. We also conducted separate analyses of each individual metric, as a secondary objective of the research.

### Assessments of gestational CVH

Gestational CVH metrics were evaluated according to the AHA recommendations [4,6]. Smoking status was self-reported in the gestational questionnaire. Pregnant women who had never smoked or stopped smoking for >12 months were regarded as having ideal metrics; those who had smoked within the preceding 12 months were regarded as having intermediate metrics, and current smokers were regarded as having poor metrics. BMI was calculated from weight and height measurements recorded at antenatal visits during gestational weeks 24–28, and blood pressure was recorded at the same visits. We defined ideal BMI as less than the top end of the range of the normal pre-pregnancy BMI (< 24 kg/m^2^) plus a cut-off for increased BMI during pregnancy; other women with higher BMIs were regarded as having non-ideal metrics. The cut-off for increased BMI during pregnancy was calculated by dividing the maximal gestational weight gain at gestational week tested by the square of the height. In accordance with the gestational weight gain standard in China [15], we added the maximal recommended gestational weight gain during the first trimester (<14 gestational weeks, 2 kg) to the maximal recommended weekly gestational weight gain for a normal pre-pregnancy BMI during the second trimester (Subtract 14 from the gestational weeks tested and then multiply by 0.48 kg/week), to calculate the maximal gestational weight gain. Regarding blood pressure, we defined < 120/< 80 mm Hg untreated as ideal; systolic blood pressure of 120–139 mm Hg, diastolic blood pressure of 80–89 mm Hg, or treated to goal as intermediate; and systolic blood pressure ≥ 140 mm Hg or diastolic blood pressure ≥90 mm Hg as poor. Total cholesterol and fasting blood glucose were measured in the biochemical laboratory of Women’s Hospital School of Medicine Zhejiang University from venous blood samples drawn during gestational weeks 24–28 after overnight fasting. The blood samples were centrifuged (3,500 rpm for 10 min at 4 °C) to extract serum and assayed using an AU400e chemistry immune analyzer (Olympus, Tokyo, Japan). Women with standard fasting blood glucose measurements for gestational diabetes mellitus (≥ 92 mg/dL or 5.1 mmol/L) [16] were regarded as having poor metrics; all other women were regarded as having ideal metrics. We did not use the values recommended by the AHA for the general population to evaluate cholesterol levels, because pregnant women often exhibit hyperlipidemia due to changes in metabolism and sex hormones [17,18]. Instead, we used the gestational total cholesterol metrics defined by the HAPO study [9]: <260 mg/dL (<6.73 mmol/L) untreated was ideal, 260–300 mg/dL (6.73–7.76 mmol/L) or treated to goal was intermediate, and ≥300 mg/dL (≥7.76 mmol/L) was poor. Sleep health was also self-reported in the gestational questionnaire. Because there are no guidelines defining risk levels for gestational sleep health, we used the AHA standard published in 2022 [5]: 7–10 h of sleep per night was ideal, 6–7 h was intermediate, and < 6 or ≥ 10 h was poor (Table S1).

Total gestational CVH scores were calculated by adding the separate CVH metrics, assigning 2 points for ideal, 1 point for intermediate, and 0 points for poor metrics, which was the primary exposure measure. To evaluate the role of total gestational CVH level better, we also compared the groups of pregnant women with all ideal metrics and those with any non-ideal metrics, and further categorized it as high CVH (all ideal metrics), moderate CVH (no poor metrics), or low CVH (one or more poor metric) [3], as secondary exposure measures.

### Assessments of adverse pregnancy outcomes

In this study, adverse maternal and neonatal outcomes were regarded as primary outcomes, and various complications were also analyzed separately.

Adverse maternal outcomes included at least one of the following complications: hypertensive disorders of pregnancy (HDP), intrahepatic cholestasis of pregnancy (ICP), fetal distress, preterm birth, and primary cesarean delivery. HDP is defined as hypertensive disorders that occur during pregnancy, including gestational hypertension, pre-eclampsia, eclampsia, chronic hypertension complicating pregnancy, and chronic hypertension with superimposed pre-eclampsia. The International Society for the Study of Hypertension in Pregnancy diagnostic criteria were used [19]. A diagnosis of ICP was based on symptoms of pruritus and elevated bile acid levels, as well as possible abnormal liver function without a secondary cause of liver dysfunction [20]. Fetal distress was diagnosed based on abnormal fetal-heart-monitor measurements and amniotic fluid status. Preterm delivery was delivery between 28 and 37 gestational weeks. Primary cesarean delivery was defined as the first cesarean delivery performed due to various obstetric indications, excluding cesarean delivery caused by a scarred uterus (including prior cesarean delivery and uterine surgery).

Adverse neonatal outcomes included at least one of the following complications: large for gestational age (LGA), small for gestational age (SGA), macrosomia, low birth weight, admission to neonatal intensive care unit (NICU), neonatal hyperbilirubinemia, neonatal respiratory diseases, and neonatal hypoglycemia. LGA and SGA were defined as a birth weight above the 90^th^ and below the 10^th^ percentile for gestational age, respectively [21]. Macrosomia was defined as a birth weight ≥ 4,000 g, whereas < 2,500 g was defined as a low birth weight. Newborns were admitted to the NICU for various reasons, which were recorded. Neonatal hyperbilirubinemia was recorded when bilirubin levels exceeded the expected newborn bilirubin profile, and neonatal hypoglycemia was recorded when blood glucose levels were < 39.6 mg/dL (2.2 mmol/L). Neonatal respiratory diseases included shortness of breath, groaning, abnormal respiratory signs, pneumonia, and respiratory distress syndrome, as well as any other respiratory-system diseases.

### Statistical analysis

Variables were compared using analysis of variance (ANOVA) and χ^2^ analyses as appropriate. *Post hoc* pairwise comparisons between groups were performed using the Bonferroni method. The absolute risk was calculated as a percentile of adverse pregnancy outcomes in each gestational CVH score category. Relative risks (RRs) with 95% confidence intervals (CIs) were calculated using modified Poisson regression with robust standard errors, adjusting for maternal age, gravidity, parity, occupation (home vs. low physical vs. medium physical vs. high physical), income (low vs. medium vs. high), education (high school and below vs. bachelor vs. postgraduate), and gestational week tested. We also performed three sensitivity analyses. In the first, we used the AHA recommendations for total cholesterol [4,6]: < 200 mg/dL (< 5.18 mmol/L) untreated was considered ideal, 200–240 mg/dL (5.18–6.21 mmol/L) or treated to goal was considered intermediate, and ≥ 240 mg/dL (≥ 6.21 mmol/L) was considered poor. In the second, we excluded sleep-health metrics because standard metrics for pregnant women have not been established. In the third, in order to assess the potential impact of missing data on the results, we included 234 pregnant women who were excluded due to data absence in the analysis using multiple imputation. Post hoc power analysis for Poisson regression using G*Power software (ver. 3.1; Franz Faul, University of Kiel, Kiel, Germany) showed that the study had sufficient power (> 80%). The calculations were based on the observed effect size obtained from the current data, with a total sample size of 2,775 and a significance level of 0.05 (two-tailed). Data were analyzed using SPSS software (ver. 22.0; IBM Corp., Armonk, NY, USA), and a *P*-value < 0.05 was considered statistically significant.

## Results

**Figure 1.**
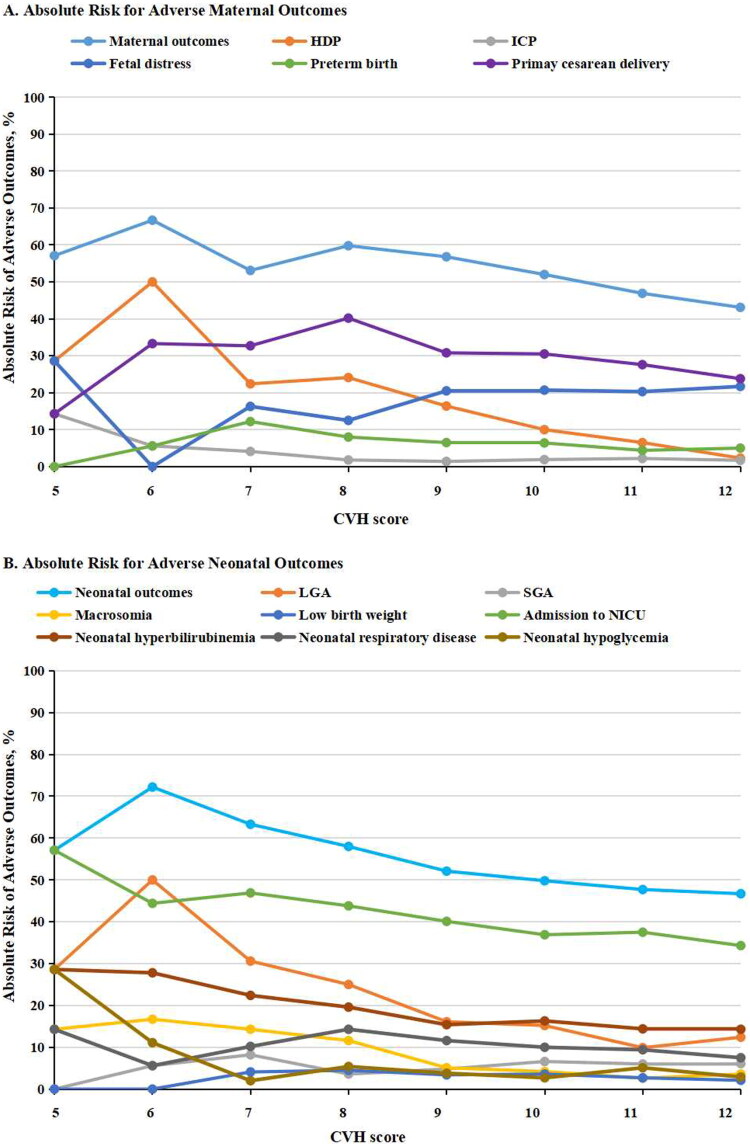
Absolute risk of adverse pregnancy outcomes according to gestational cardiovascular health score. Absolute risk of adverse maternal (A) and neonatal (B) outcomes was calculated as (No. of women with adverse outcome/No. of women in gestational cardiovascular health [CVH] score category) × 100. The symbols represent the absolute risk in each CVH score category. Adverse maternal outcomes include the following: hypertensive disorders of pregnancy (HDP), intrahepatic cholestasis of pregnancy (ICP), fetal distress, preterm birth, and primary cesarean delivery. Adverse neonatal outcomes include the following: large for gestational age (LGA), small for gestational age (SGA), macrosomia, low birth weight, admission to neonatal intensive care unit (NICU), neonatal hyperbilirubinemia, neonatal respiratory disease, and neonatal hypoglycemia.

The 2,775 participants’ characteristics are shown in Table 1. The mean maternal age was 30.88 ± 4.00 years, and the mean number of gestational weeks when questionnaire and CVH metrics data were collected was 25.27 ± 1.42 weeks. In total, 1,001 (36.07%) pregnant women had high CVH, 1,002 (36.11%) had moderate CVH, and 772 (27.82%) had low CVH. The mean gestational CVH score of the 2,775 pregnant women was 10.72 ± 1.34 out of 12. Maternal age, gravidity, and parity were highest in the low-CVH group, although there were no statistically significant group differences in some of the values. Similarly, pregnant women who stayed at home, had lower incomes, or had lower education levels were comparatively more common in the low-CVH group. The distributions of gestational CVH metrics and gestational CVH scores differed significantly among the three CVH-level groups (*p* < 0 .05). Figure 1 shows the absolute risk of adverse pregnancy outcomes according to gestational CVH score, without adjusting for confounding factors. The incidence rates of adverse maternal and neonatal outcomes generally decreased with increasing gestational CVH score.

**Table 1.** Participant characteristics according to gestational cardiovascular health status.

	Total (*N* = 2,775)	High CVH (All ideal metrics) (*n* = 1,001)	Moderate CVH (0 poor metrics) (*n* = 1,002)	Low CVH (≥ 1 poor metric) (*n* = 772)	*P*
Maternal age (years)	30.88 ± 4.00	30.84 ± 3.77	30.71 ± 3.87	31.15 ± 4.44	0.065
Gravidity	1.83 ± 1.04	1.79 ± 1.00	1.76 ± 1.00	1.96 ± 1.14*^#^	<0.001
Parity	0.32 ± 0.51	0.33 ± 0.51	0.28 ± 0.48	0.36 ± 0.53^#^	0.010
Occupation					<0.001
Home	282 (10.2)	91 (9.1)	88 (8.8)	103 (13.3)*^#^	
Low physical	1,976 (71.2)	718 (71.7)	752 (75.0)	506 (65.5)*^#^	
Medium physical	507 (18.3)	190 (19.0)	161 (16.1)	156 (20.2)	
High physical	10 (0.4)	2 (0.2)	1 (0.1)	7 (0.9)^#^	
Income					<0.001
Low	191 (6.9)	59 (5.9)	54 (5.4)	78 (10.1)*^#^	
Medium	907 (32.7)	318 (31.8)	322 (32.1)	267 (34.6)	
High	1,677 (60.4)	624 (62.3)	626 (62.5)	427 (55.3)*^#^	
Education					<0.001
High school and below	838 (30.2)	250 (25.0)	284 (28.3)	304 (39.4)*^#^	
Bachelor	1,455 (52.4)	558 (55.7)	535 (53.4)	362 (46.9)*^#^	
Postgraduate	482 (17.4)	193 (19.3)	183 (18.3)	106 (13.7)*^#^	
Gestational week tested	25.27 ± 1.42	25.14 ± 1.40	25.30 ± 1.45*	25.39 ± 1.40*	0.001
Birth gestational week	38.84 ± 1.34	38.94 ± 1.34	38.83 ± 1.33	38.71 ± 1.36*	0.002
Birth weight (g)	3,275.44 ± 430.82	3,263.81 ± 412.28	3,251.58 ± 405.60	3,321.48 ± 480.31*^#^	0.002
Apgar score 1 min	9.82 ± 0.71	9.86 ± 0.68	9.80 ± 0.70	9.81 ± 0.78	0.218
Apgar score 5 min	9.96 ± 0.48	9.96 ± 0.52	9.97 ± 0.36	9.94 ± 0.56	0.508
Newborn sex					0.598
Female	1,307 (47.1)	484 (48.4)	467 (46.6)	356 (46.1)	
Male	1,468 (52.9)	517 (51.6)	535 (53.4)	416 (53.9)	
Smoking grade					<0.001
Poor	11 (0.4)	0 (0.0)	0 (0.0)	11 (1.4)*^#^	
Intermediate	28 (1.0)	0 (0.0)	19 (1.9)*	9 (1.2)*	
Ideal	2,736 (98.6)	1,001 (100)	983 (98.1)*	752 (97.4)*	
Blood pressure grade					<0.001
Poor	32 (1.2)	0 (0.0)	0 (0.0)	32 (4.1)*^#^	
Intermediate	670 (24.1)	0 (0.0)	421 (42.0)*	249 (32.3)*^#^	
Ideal	2,073 (74.7)	1,001 (100)	581 (58.0)*	491 (63.6)*^#^	
Body mass index grade					<0.001
Poor	350 (12.6)	0 (0.0)	0 (0.0)	350 (45.3)*^#^	
Ideal	2,425 (87.4)	1,001 (100)	1,002 (100)	422 (54.7)*^#^	
Fasting blood glucose grade					<0.001
Poor	121 (4.4)	0 (0.0)	0 (0.0)	121 (15.7)*^#^	
Ideal	2,654 (95.6)	1,001 (100)	1,002 (100)	651 (84.3)*^#^	
Total cholesterol grade					<0.001
Poor	192 (6.9)	0 (0.0)	0 (0.0)	192 (24.9)*^#^	
Intermediate	561 (20.2)	0 (0.0)	459 (45.8)*	102 (13.2)*^#^	
Ideal	2,022 (72.9)	1,001 (100)	543 (54.2)*	478 (61.9)*^#^	
Sleep health grade					<0.001
Poor	198 (7.1)	0 (0.0)	0 (0.0)	198 (25.6)*^#^	
Intermediate	486 (17.5)	0 (0.0)	366 (36.5)*	120 (15.5)*^#^	
Ideal	2,091 (75.4)	1,001 (100)	636 (63.5)*	454 (58.8)*	
Gestational CVH score	10.72 ± 1.34	12.00 ± 0.00	10.74 ± 0.50*	9.04 ± 1.08*^#^	<0.001

Abbreviation: CVH, cardiovascular health.

Data are expressed as means ± standard deviations or numbers (percentages). All *post hoc* group comparisons were performed using the Bonferroni method. *p < 0.05 vs. Ideal; ^#^*p* < 0.05 vs. Intermediate.

Associations between gestational CVH and adverse maternal outcomes are shown in Table 2. Overall, higher gestational CVH scores were significantly correlated with lower odds of adverse maternal outcomes (RR, 0.93; 95% CI, 0.90–0.95). Each one-point increase in the gestational CVH score was associated with a 36% lower risk of HDP (RR, 0.64; 95% CI, 0.60–0.68) and a 7% lower risk of primary cesarean delivery (RR, 0.93; 95% CI, 0.89–0.96). Furthermore, compared to having low or moderate gestational CVH, high gestational CVH was associated with a 15% lower risk of adverse maternal outcomes (RR, 0.85; 95% CI, 0.78–0.92). Regarding the different adverse maternal outcomes, the odds of HDP (RR, 0.15; 95% CI, 0.10–0.22) and primary cesarean delivery (RR, 0.76; 95% CI, 0.66–0.88) were lower in pregnant women with high gestational CVH, whereas the odds of ICP, preterm birth and fetal distress were not significantly altered.

**Table 2.** Associations between gestational cardiovascular health and adverse maternal outcomes.

	Events^a^	RR (95% CI)^b^	*P*
**Maternal outcomes**			
CVH score	1341 (48.3)	0.93 (0.90–0.95)	<0.001
All ideal^c^ (vs. any non-ideal^d^)	431 (43.1) vs. 910 (51.3)	0.85 (0.78–0.92)	<0.001
CVH grade			<0.001
Low CVH^e^	422 (54.7)	Reference	–
Moderate CVH^f^	488 (48.7)	0.88 (0.80–0.96)	0.003
High CVH^c^	431 (43.1)	0.79 (0.72–0.87)	<0.001
Hypertensive disorders of pregnancy			
CVH score	223 (8.1)	0.64 (0.60–0.68)	<0.001
All ideal (vs. any non-ideal)	23 (2.3) vs. 200 (11.3)	0.21 (0.14–0.32)	<0.001
CVH grade			<0.001
Low CVH	124 (16.1)	Reference	–
Moderate CVH	76 (7.6)	0.48 (0.37–0.63)	<0.001
High CVH	23 (2.3)	0.15 (0.10–0.22)	<0.001
Intrahepatic cholestasis of pregnancy			
CVH score	54 (1.9)	0.91 (0.75–1.12)	0.378
All ideal (vs. any non-ideal)	17 (1.7) vs. 37 (2.1)	0.82 (0.47–1.44)	0.491
CVH grade			0.674
Low CVH	15 (1.9)	Reference	–
Moderate CVH	22 (2.2)	1.21 (0.62–2.35)	0.584
High CVH	17 (1.7)	0.91 (0.46–1.83)	0.800
Fetal distress			
CVH score	566 (20.4)	1.05 (0.99–1.12)	0.080
All ideal (vs. any non-ideal)	217 (21.7) vs. 349 (19.7)	1.10 (0.95–1.28)	0.219
CVH grade			0.451
Low CVH	145 (18.8)	Reference	–
Moderate CVH	204 (20.4)	1.03 (0.85–1.24)	0.779
High CVH	217 (21.7)	1.12 (0.92–1.35)	0.252
Preterm birth			
CVH score	153 (5.5)	0.92 (0.83–1.02)	0.122
All ideal (vs. any non-ideal)	50 (5.0) vs. 103 (5.8)	0.91 (0.66–1.26)	0.554
CVH grade			0.416
Low CVH	53 (6.9)	Reference	–
Moderate CVH	50 (5.0)	0.80 (0.55–1.17)	0.253
High CVH	50 (5.0)	0.81 (0.55–1.18)	0.263
Primary cesarean delivery			
CVH score	769 (27.7)	0.93 (0.89–0.96)	<0.001
All ideal (vs. any non-ideal)	238 (23.8) vs. 531 (29.9)	0.82 (0.72–0.93)	0.002
CVH grade			0.001
Low CVH	246 (31.8)	Reference	–
Moderate CVH	285 (28.4)	0.87 (0.77–1.00)	0.046
High CVH	238 (23.8)	0.76 (0.66–0.88)	<0.001

Abbreviations: RR, relative risk; CI, confidence interval; CVH, cardiovascular health.

^a^ Events were expressed as numbers (percentages).

^b^ All analyses were adjusted for maternal age, gravidity, parity, occupation, income, education, and gestational week tested.

^c^ Pregnant women with all ideal metrics.

^d^ Pregnant women with one non-ideal metric.

^e^ Pregnant women with at least one poor metric.

^f^ Pregnant women with non-ideal metrics but no poor metrics.

Associations between gestational CVH and adverse neonatal outcomes are shown in Table 3. Each one-point increase in the gestational CVH score was associated with a 4% lower risk of adverse neonatal outcomes (RR, 0.96; 95% CI, 0.93–0.98). Regarding the different adverse neonatal outcomes, a higher gestational CVH score was significantly associated with lower odds of LGA (RR, 0.84; 95% CI, 0.79–0.89), macrosomia (RR, 0.76; 95% CI, 0.67–0.85), admission to the NICU (RR, 0.95; 95% CI, 0.92–0.99), neonatal hyperbilirubinemia (RR, 0.93; 95% CI, 0.88–1.00), and neonatal respiratory disease (RR, 0.91; 95% CI 0.84–0.99). Moreover, pregnant women with high CVH (all ideal metrics) were less likely to develop adverse neonatal outcomes (RR, 0.88; 95% CI, 0.80–0.96), including LGA (RR, 0.65; 95% CI, 0.52–0.81), macrosomia (RR, 0.52; 95% CI, 0.34–0.79), admission to the NICU (RR, 0.85; 95% CI, 0.76–0.97), or neonatal respiratory disease (RR, 0.67; 95% CI, 0.50–0.90) than those with low CVH based on one or more poor metrics.

**Table 3.** Associations between gestational cardiovascular health and adverse neonatal outcomes.

	Events^a^	RR (95% CI)^b^	*P*
**Neonatal outcomes**			
CVH score	1360 (49.0)	0.96 (0.93–0.98)	0.001
All ideal^c^ (vs. any non-ideal^d^)	467 (46.7) vs. 893 (50.3)	0.94 (0.86–1.01)	0.106
CVH grade			0.014
Low CVH^e^	417 (54.0)	Reference	–
Moderate CVH^f^	476 (47.5)	0.89 (0.81–0.98)	0.015
High CVH^c^	467 (46.7)	0.88 (0.80–0.96)	0.006
Large for gestational age			
CVH score	381 (13.7)	0.84 (0.79–0.89)	< 0.001
All ideal (vs. any non-ideal)	124 (12.4) vs. 257 (14.5)	0.85 (0.70–1.04)	0.114
CVH grade			< 0.001
Low CVH	149 (19.3)	Reference	–
Moderate CVH	108 (10.8)	0.58 (0.46–0.74)	< 0.001
High CVH	124 (12.4)	0.65 (0.52–0.81)	< 0.001
Small for gestational age			
CVH score	165 (5.9)	1.04 (0.93–1.17)	0.464
All ideal (vs. any non-ideal)	61 (6.1) vs. 104 (5.9)	1.07 (0.79–1.45)	0.680
CVH grade			0.772
Low CVH	42 (5.4)	Reference	–
Moderate CVH	62 (6.2)	1.12 (0.77–1.65)	0.555
High CVH	61 (6.1)	1.14 (0.78–1.68)	0.498
Macrosomia			
CVH score	117 (4.2)	0.76 (0.67–0.85)	<0.001
All ideal (vs. any non-ideal)	36 (3.6) vs. 81 (4.6)	0.78 (0.53–1.15)	0.201
CVH grade			<0.001
Low CVH	53 (6.9)	Reference	–
Moderate CVH	28 (2.8)	0.41 (0.26–0.65)	<0.001
High CVH	36 (3.6)	0.52 (0.34–0.79)	0.002
Low birth weight			
CVH score	78 (2.8)	0.91 (0.79–1.05)	0.181
All ideal (vs. any non-ideal)	21 (2.1) vs. 57 (3.2)	0.70 (0.43–1.14)	0.155
CVH grade			0.338
Low CVH	27 (3.5)	Reference	–
Moderate CVH	30 (3.0)	0.90 (0.53–1.52)	0.692
High CVH	21 (2.1)	0.66 (0.37–1.17)	0.152
Admission to neonatal intensive care unit			
CVH score	1026 (37.0)	0.95 (0.92–0.99)	0.005
All ideal (vs. any non-ideal)	343 (34.3) vs. 683 (38.5)	0.90 (0.81–1.00)	0.048
CVH grade			0.041
Low CVH	314 (40.7)	Reference	–
Moderate CVH	369 (36.8)	0.91 (0.81–1.03)	0.126
High CVH	343 (34.3)	0.85 (0.76–0.97)	0.011
Neonatal hyperbilirubinemia			
CVH score	424 (15.3)	0.93 (0.88–1.00)	0.034
All ideal (vs. any non-ideal)	143 (14.3) vs. 281 (15.8)	0.92 (0.76–1.11)	0.366
CVH grade			0.235
Low CVH	134 (17.4)	Reference	–
Moderate CVH	147 (14.7)	0.86 (0.69–1.06)	0.158
High CVH	143 (14.3)	0.84 (0.67–1.05)	0.124
Neonatal respiratory disease			
CVH score	257 (9.3)	0.91 (0.84–0.99)	0.019
All ideal (vs. any non-ideal)	75 (7.5) vs. 182 (10.3)	0.75 (0.58–0.97)	0.026
CVH grade			0.026
Low CVH	90 (11.7)	Reference	–
Moderate CVH	92 (9.2)	0.81 (0.62–1.08)	0.146
High CVH	75 (7.5)	0.67 (0.50–0.90)	0.007
Neonatal hypoglycemia			
CVH score	104 (3.7)	0.91 (0.79–1.05)	0.185
All ideal (vs. any non-ideal)	29 (2.9) vs. 75 (4.2)	0.71 (0.46–1.08)	0.107
CVH grade			0.262
Low CVH	34 (4.4)	Reference	–
Moderate CVH	41 (4.1)	0.96 (0.61–1.50)	0.859
High CVH	29 (2.9)	0.69 (0.42–1.13)	0.141

Abbreviations: RR, relative risk; CI, confidence interval; CVH, cardiovascular health.

^a^ Events were expressed as numbers (percentages).

^b^ All analyses were adjusted for maternal age, gravidity, parity, occupation, income, education, and gestational week tested.

^c^ Pregnant women with all ideal metrics.

^d^ Pregnant women with one non-ideal metric.

^e^ Pregnant women with at least one poor metric.

^f^ Pregnant women with non-ideal metrics but no poor metrics.

**Figure 2.**
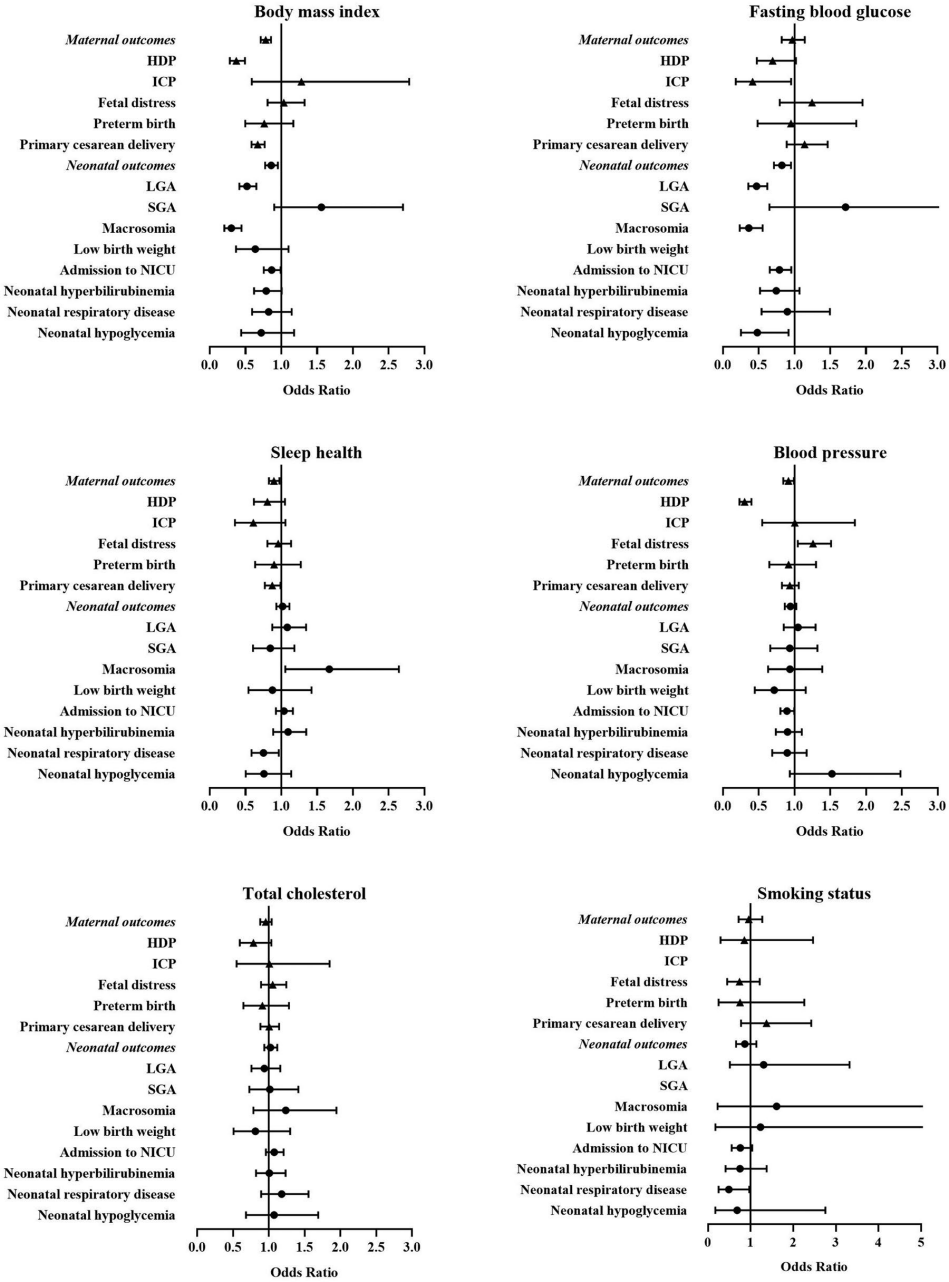
Associations between gestational cardiovascular health metrics and adverse maternal and/or neonatal outcomes. All analyses were adjusted for maternal age, gravidity, parity, occupation, income, education, gestational week tested, and other gestational cardiovascular health (CVH) metrics. Gestational CVH metrics with ideal status were compared with those with non-ideal (including intermediate and poor) status. Error bars represent 95% confidence intervals. Adverse maternal outcomes include the following: hypertensive disorders of pregnancy (HDP), intrahepatic cholestasis of pregnancy (ICP), fetal distress, preterm birth, and primary cesarean delivery. Adverse neonatal outcomes include the following: large for gestational age (LGA), small for gestational age (SGA), macrosomia, low birth weight, admission to neonatal intensive care unit (NICU), neonatal hyperbilirubinemia, neonatal respiratory disease, and neonatal hypoglycemia.

We also evaluated the associations between separate CVH metrics and adverse pregnancy outcomes (Figure 2). An ideal BMI (vs. non-ideal, including intermediate and poor status) was associated with a 22% lower risk of adverse maternal outcomes (RR, 0.78; 95% CI, 0.71–0.86) and a 14% lower risk of adverse neonatal outcomes (RR, 0.86; 95% CI, 0.78–0.95). The relationship between ideal fasting blood glucose and adverse maternal outcomes was not statistically significant (RR, 0.97; 95% CI, 0.82–1.15), though it was significantly associated with a lower risk of adverse neonatal outcomes (RR, 0.83; 95% CI, 0.72–0.95). The risks of adverse maternal outcomes were also lower for those with ideal blood pressure (RR, 0.92; 95% CI, 0.85–0.99) and ideal sleep health (RR, 0.90; 95% CI, 0.83–0.97), whereas the odds of adverse neonatal outcomes were not significantly altered (RR, 0.94; 95% CI, 0.87–1.03; RR, 1.02; 95% CI, 0.93–1.11). There was no significant relationship between total cholesterol and adverse maternal outcomes (RR, 0.96; 95% CI, 0.88–1.04) or neonatal outcomes (RR, 1.02; 95% CI, 0.94–1.12), or between smoking status and adverse maternal outcomes (RR, 0.96; 95% CI, 0.72–1.27) or neonatal outcomes (RR, 0.87; 95% CI, 0.66–1.14). With the exceptions of total cholesterol, our results indicate that, for each CVH metric, ideal status significantly influenced at least one adverse maternal or neonatal outcome.

In a sensitivity analysis that used the total-cholesterol grades recommended by the AHA, the participant characteristics were slightly different. Here, the mean gestational CVH score was 9.74 ± 1.36, 6.09% of pregnant women had high CVH, 34.38% had moderate CVH, and 59.53% had low CVH. The associations between gestational CVH scores and adverse pregnancy outcomes were similar to those described in the primary analysis, but the associations were somewhat different for total gestational CVH levels. These results indicated that the risk of adverse neonatal outcomes was significantly lower in pregnant women with high gestational CVH than in women with low or moderate gestational CVH (RR, 0.78; 95% CI, 0.65–0.95; Table S2). In a sensitivity analysis that excluded the sleep-health metric, the associations between gestational CVH scores and adverse pregnancy outcomes were also similar to those described in the primary analysis, except for a slightly different association between total gestational CVH level and neonatal outcomes (RR, 0.91; 95% CI, 0.84–0.98; Table S3). In addition, a total of 234 pregnant women were excluded due to missing data. We compared the characteristics of this group to those with complete data. It was found that there were no significant differences in the participant characteristics, except for income and birth weight (Table S4). The sensitivity analysis using multiple imputation showed that the associations between gestational CVH and adverse pregnancy outcomes were basically consistent with those described in the primary analysis (Table S5).

## Discussion

In this study, we integrated six metrics (smoking status, BMI, blood pressure, total cholesterol, fasting blood glucose, and sleep health) to evaluate CVH during pregnancy; we found that gestational CVH scores were inversely correlated with adverse pregnancy outcomes. Pregnant women with high CVH had a considerably lower risk of adverse maternal and neonatal outcomes than those with low CVH. However, 63.93% of the pregnant women in this Chinese cohort did not achieve a high level of CVH.

To our knowledge, this is the first study to describe gestational CVH characteristics including sleep health and investigate the relationships between these characteristics and adverse maternal and neonatal outcomes in Chinese women. A previous study from the United States by Perak, A.M et al. based on National Health and Nutrition Examination Surveys from 1999 to 2004, found that CVH levels were significantly lower among pregnant than among non-pregnant women, with only 4.6% of pregnant women having a high level of CVH [4]. The Generation R Study found that 18.8% of pregnant White women had a high level of CVH, and better CVH in early pregnancy was associated with reduced carotid intima‐media thickness [22]. Similar results were observed in another cohort, in which better maternal CVH during pregnancy (32.8%) was significantly correlated with better offspring CVH in early adolescence [9]. A study based on the HAPO cohort linked gestational CVH level with adverse pregnancy outcomes, first established a preliminary CVH standard for pregnant women, and served as an important point of reference for our study. The study authors showed that pregnant women with more favorable gestational CVH (36.3%) had a significantly lower risk of adverse pregnancy outcomes such as pre-eclampsia, primary cesarean delivery, and LGA, which was similar with our study [3]. However, that study focused on European and American populations and had relatively few outcome measures. Gao et al. [23] found in a China birth cohort that poor gestational CVH in the first trimester (48.6%) was associated with a significantly increased risk of adverse pregnancy outcomes, but this study only included five CVH metrics and did not assess sleep health. Another cross-sectional study evaluated the status of gestational CVH based on Life’s Essential 8 and found that only a small proportion (12.84%) had high CVH, which was associated with lower risk of birth outcomes in newborns [24]. Notably, our study is the first to incorporate sleep health into gestational CVH evaluations as a new metric, and to assess associations with adverse maternal and neonatal outcomes comprehensively. Our findings indicate that CVH influences multiple adverse pregnancy outcomes in Chinese women and highlight the importance of optimizing CVH during pregnancy to improve the short- and long-term health of pregnant women and their offspring. However, we can also observe that the proportion of pregnant women with high CVH varies in different studies. This might be due to the fact that the current standards for assessing gestational CVH have not yet been determined, as well as the differences in the study population and the assessment time. In the future, we can conduct larger-scale longitudinal cohort studies to determine the appropriate standards for assessing gestational CVH in China and even around the world.

Sleep health is a new CVH metric that is particularly important because pregnant women frequently report insomnia symptoms, with an incidence as high as 38.2% [25]. Poor sleep health during pregnancy may produce oxidative stress, inflammatory responses, and hormonal imbalances, which can have negative effects on both the mother and her fetus [26,27]. In our study, ideal sleep health was associated with a 10% lower risk of adverse maternal outcomes and a 25% lower risk of neonatal respiratory diseases. Similar to the results of our study, other research has also shown that sleep disturbance during pregnancy may increase the risk of cesarean delivery [28,29]. Notably, we found that ideal sleep health was associated with a higher risk of macrosomia; ideal sleep health can regulate the circadian rhythms of pregnant women, encouraging maternal melatonin to cross the placenta and thereby promoting the growth and development of the fetus [30,31]. Considering the clinical application and the definition of healthy sleep in the AHA recommendations, our CVH sleep-health assessments included sleep duration but not the effects of sleep patterns, sleep quality, or other factors. This may also partly account for our surprising observation linking ideal sleep duration with macrosomia. As reported previously, BMI was a particularly important factor, and a high BMI was strongly associated with adverse pregnancy outcomes [32,33]. Similarly, we found that hyperglycemia [34], hypertension [35], and maternal smoking [36] were also associated with adverse pregnancy outcomes, while hyperlipidemia were not. It was inconsistent with previous literature reports [37], which may be due to different gestational total cholesterol metrics. Overall, our data highlighted similar associations between the separate CVH metrics and adverse pregnancy outcomes, demonstrating that a combined evaluation of CVH can be used to assess pregnant women. Furthermore, total gestational CVH scores correlated more strongly with maternal and neonatal outcomes than did CVH levels (all ideal metrics vs. no poor metrics or at least one poor metric), validating the more comprehensive approach. Clearly, improvement of gestational CVH can reduce the likelihood of adverse pregnancy outcomes.

To the best of our knowledge, our study is the first comprehensively investigating gestational CVH in relation to adverse maternal and neonatal outcomes in pregnant Chinese women and include the new metric of sleep health in evaluations of gestational CVH. Our results imply that CVH evaluations and interventions are needed for pregnant women in China. In general, gestational CVH can be improved and optimized by health care and interventions before and during pregnancy. Reasonable education can make pregnant women and their families aware of the harm of low CVH to both mothers and their offspring. In terms of treatment goals, achieving all ideal metrics (12 points of gestational CVH score) is undoubtedly the ultimate objective, but this may not be accomplished within a short period of time. During this process, improvement in any of the metrics can be beneficial in reducing the risk of adverse pregnancy outcomes. For pregnant women with a low CVH score, we recommend prioritizing the elimination of poor metrics. Even merely reaching the intermediate level can significantly improve the pregnancy outcomes. Meanwhile, more studies are needed in the future to assist in completing the gestational CVH evaluation metrics and standards. Studies on CVH assessment at multiple time points during pregnancy can provide us with more comprehensive information. Clinical trials can also be utilized to explore better clinical intervention measures. These dual efforts at the individual and social levels will be of great significance for improving health in entire life cycle.

However, our study had some limitations. First, we lacked data on diet and physical activity, and these factors may affect the CVH evaluations of pregnant women; this data deficiency needs to be improved in future studies. Nonetheless, diet and exercise data may be reflected, in part, by BMI measurements. Second, our data were mainly from a single time window during the second trimester of pregnancies. However, pregnancy is a dynamic physiological process. Our study may not capture the full trajectory of CVH influence on pregnancy outcomes and affect the interpretation and generalizability of the results. In the future, a longitudinal tracking study of CVH throughout the entire pregnancy period may provide a more complete understanding. Third, the evaluation of sleep health was based on self-reported sleep duration, lacking other relevant indicators. Professional instruments (e.g. Pittsburgh Sleep Quality Index) in future studies may be able to provide a more comprehensive assessment of gestational sleep health and explain its association with adverse outcomes. Finally, the criteria for CVH in pregnant Chinese women need to be optimized. Differences in cultural and lifestyle factors, as well as differences among racial groups, may impact observations, and large-scale studies are needed to ensure that the CVH criteria used are applicable to pregnant women in China.

## Conclusions

In conclusion, high gestational CVH based on six separate metrics significantly correlated with lower risks of adverse maternal and neonatal outcomes in Chinese women. However, approximately two-thirds of pregnant Chinese women do not achieve a high level of CVH. Timely clinical evaluation of CVH in pregnant women, comprehensive management and improvement of these six metrics levels, may help reduce the risk of adverse pregnancy outcomes.

## Supplementary Material

Supplementary Files_Clean.docx

## Data Availability

The data that support the findings of this study are available from the corresponding author LZX upon reasonable request.
