# Supplementary material for: Gestational cardiovascular health and adverse pregnancy outcomes: a prospective cohort study in China
Source: Ann Med. 2026 Jul 26;58(1):2703887. doi: 10.1080/07853890.2026.2703887 (PMC13410539; doi:10.1080/07853890.2026.2703887)
Supplement: Supplementary Files_Clean.docx [file IANN_A_2703887_SM3035.docx]

**Supplementary Table 1. Quantification of sleep health using the American Heart Association standard**

| **Points** | **Level** |
| --- | --- |
| 100 | 7–9 h |
| 90 | 9–10 h |
| 70 | 6–7 h |
| 40 | 5–6 or ≥ 10 h |
| 20 | 4–5 h |
| 0 | < 4 h |

A presidential advisory from the American Heart Association published in 2022^4^ recommended that all cardiovascular health metrics should be scaled from 0 to 100 points: overall CVH scores of 80–100 points are considered high CVH, scores of 50–79 points are equivalent to moderate CVH, and scores of 0–49 points correspond to low CVH. In addition, studies have shown that tiredness and fatigue are the most common symptoms during pregnancy, and total sleep duration during pregnancy tends to be longer than before pregnancy [38, 39]. In our study, we equated 100 and 90 points (7–10 h) with ideal sleep health, 70 points (6–7 h) with intermediate sleep health, and 40, 20, or 0 points (< 6 or ≥ 10 h) with poor sleep health.

**Supplementary Table 2. Associations between gestational cardiovascular health and adverse maternal and/or neonatal outcomes based on total cholesterol grade of the Journal of the American Heart Association**

|  | **RR (95% CI)^a^** | **RR (95% CI)^a^** |
| --- | --- | --- |
|  | Maternal outcomes | Neonatal outcomes |
| CVH score | 0.93 (0.91–0.96) | 0.96 (0.93–0.98) |
| All ideal^b^  (vs. Any non-ideal^c^) | 0.75 (0.62–0.92) | 0.78 (0.65–0.95) |
| High CVH^b^  (vs. Low CVH^d^) | 0.73 (0.60–0.90) | 0.78 (0.64–0.95) |
| Moderate CVH^e^  (vs. Low CVH) | 0.93 (0.85–1.00) | 0.99 (0.92–1.08) |
|  | Hypertensive disorders of pregnancy | Large for gestational age |
| CVH score | 0.64 (0.60–0.68) | 0.84 (0.79–0.90) |
| All ideal  (vs. Any non-ideal) | 0.07 (0.01–0.51) | 0.68 (0.42–1.08) |
| High CVH  (vs. Low CVH) | 0.06 (0.08–0.41) | 0.62 (0.38–0.99) |
| Moderate CVH  (vs. Low CVH) | 0.45 (0.33–0.62) | 0.75 (0.61–0.92) |
|  | Intrahepatic cholestasis of pregnancy | Small for gestational age |
| CVH score | 0.88 (0.73–1.06) | 1.11 (0.99–1.25) |
| All ideal  (vs. Any non-ideal) | 0.31 (0.04–2.25) | 1.25 (0.71–2.19) |
| High CVH  (vs. Low CVH) | 0.31 (0.04–2.24) | 1.45 (0.81–2.59) |
| Moderate CVH  (vs. Low CVH) | 0.97 (0.56–1.70) | 1.44 (1.06–1.96) |
|  | Fetal distress | Macrosomia |
| CVH score | 1.06 (1.00–1.12) | 0.76 (0.68–0.84) |
| All ideal  (vs. Any non-ideal) | 0.97 (0.71–1.32) | 0.13 (0.02–0.95) |
| High CVH  (vs. Low CVH) | 1.01 (0.74–1.40) | 0.12 (0.02–0.87) |
| Moderate CVH  (vs. Low CVH) | 1.14 (0.98–1.33) | 0.77 (0.52–1.13) |
|  | Preterm birth | Low birth weight |
| CVH score | 0.93 (0.84–1.03) | 0.92 (0.80–1.06) |
| All ideal  (vs. Any non-ideal) | 0.88 (0.44–1.78) | 0.88 (0.33–2.36) |
| High CVH  (vs. Low CVH) | 0.83 (0.41–1.69) | 0.82 (0.30–2.22) |
| Moderate CVH  (vs. Low CVH) | 0.84 (0.59–1.17) | 0.81 (0.50–1.32) |
|  | Primary cesarean delivery | Admission to neonatal intensive care unit |
| CVH score | 0.93 (0.89–0.97) | 0.95 (0.92–0.99) |
| All ideal  (vs. Any non-ideal) | 0.86 (0.65–1.14) | 0.79 (0.62–1.00) |
| High CVH  (vs. Low CVH) | 0.83 (0.62–1.10) | 0.78 (0.62–1.00) |
| Moderate CVH  (vs. Low CVH) | 0.89 (0.78–1.00) | 0.99 (0.89–1.10) |
|  | - | Neonatal hyperbilirubinemia |
| CVH score | - | 0.94 (0.88–1.01) |
| All ideal  (vs. Any non-ideal) | - | 0.80 (0.53–1.21) |
| High CVH  (vs. Low CVH) | - | 0.80 (0.53–1.22) |
| Moderate CVH  (vs. Low CVH) | - | 1.01 (0.83–1.22) |
|  | - | Neonatal respiratory disease |
| CVH score | - | 0.90 (0.83–0.97) |
| All ideal  (vs. Any non-ideal) | - | 0.71 (0.39–1.27) |
| High CVH  (vs. Low CVH) | - | 0.68 (0.37–1.23) |
| Moderate CVH  (vs. Low CVH) | - | 0.89 (0.69–1.15) |
|  | - | Neonatal hypoglycemia |
| CVH score | - | 0.93 (0.81–1.07) |
| All ideal  (vs. Any non-ideal) | - | 0.81 (0.34–1.97) |
| High CVH  (vs. Low CVH) | - | 0.79 (0.32–1.94) |
| Moderate CVH  (vs. Low CVH) | - | 0.93 (0.62–1.40) |

^a^All analyses were adjusted for maternal age, gravidity, parity, occupation, income, education, and gestational week tested. ^b^Pregnant women with all ideal metrics. ^c^Pregnant women with one non-ideal metric. ^d^Pregnant women with at least one poor metric. ^e^Pregnant women with non-ideal metrics but no poor metrics.

**Supplementary Table 3. Associations between gestational cardiovascular health and adverse maternal and/or neonatal outcomes, excluding the sleep health metric**

|  | **RR (95% CI)^a^** | **RR (95% CI)^a^** |
| --- | --- | --- |
|  | Maternal outcomes | Neonatal outcomes |
| CVH score | 0.93 (0.90–0.95) | 0.95 (0.92–0.97) |
| All ideal^b^  (vs. Any non-ideal^c^) | 0.86 (0.80–0.93) | 0.91 (0.84–0.98) |
| High CVH^b^  (vs. Low CVH^d^) | 0.80 (0.73–0.87) | 0.87 (0.79–0.95) |
| Moderate CVH^e^  (vs. Low CVH) | 0.88 (0.80–0.96) | 0.91 (0.83–1.01) |
|  | Hypertensive disorders of pregnancy | Large for gestational age |
| CVH score | 0.61 (0.57–0.65) | 0.80 (0.75–0.86) |
| All ideal  (vs. Any non-ideal) | 0.20 (0.14–0.28) | 0.76 (0.63–0.92) |
| High CVH  (vs. Low CVH) | 0.14 (0.10–0.21) | 0.56 (0.45–0.69) |
| Moderate CVH  (vs. Low CVH) | 0.52 (0.40–0.69) | 0.53 (0.41–0.68) |
|  | Intrahepatic cholestasis of pregnancy | Small for gestational age |
| CVH score | 0.98 (0.77–1.26) | 1.09 (0.94–1.25) |
| All ideal  (vs. Any non-ideal) | 1.08 (0.64–1.83) | 1.22 (0.91–1.64) |
| High CVH  (vs. Low CVH) | 1.25 (0.62–2.55) | 1.34 (0.90–2.00) |
| Moderate CVH  (vs. Low CVH) | 1.28 (0.59–2.77) | 1.17 (0.75–1.81) |
|  | Fetal distress | Macrosomia |
| CVH score | 1.08 (1.01–1.16) | 0.68 (0.60–0.77) |
| All ideal  (vs. Any non-ideal) | 1.11 (0.96–1.28) | 0.60 (0.41–0.87) |
| High CVH  (vs. Low CVH) | 1.17 (0.96–1.43) | 0.38 (0.25–0.57) |
| Moderate CVH  (vs. Low CVH) | 1.09 (0.88–1.36) | 0.35 (0.22–0.57) |
|  | Preterm birth | Low birth weight |
| CVH score | 0.91 (0.82–1.01) | 0.91 (0.78–1.06) |
| All ideal  (vs. Any non-ideal) | 0.74 (0.54–1.01) | 0.69 (0.44–1.08) |
| High CVH  (vs. Low CVH) | 0.65 (0.45–0.94) | 0.71 (0.41–1.24) |
| Moderate CVH  (vs. Low CVH) | 0.78 (0.53–1.17) | 1.06 (0.60–1.87) |
|  | Primary cesarean delivery | Admission to neonatal intensive care unit |
| CVH score | 0.93 (0.89–0.97) | 0.94 (0.91–0.98) |
| All ideal  (vs. Any non-ideal) | 0.88 (0.78–0.98) | 0.87 (0.78–0.96) |
| High CVH  (vs. Low CVH) | 0.78 (0.68–0.89) | 0.84 (0.75–0.95) |
| Moderate CVH  (vs. Low CVH) | 0.81 (0.70–0.94) | 0.96 (0.84–1.09) |
|  | - | Neonatal hyperbilirubinemia |
| CVH score | - | 0.91 (0.85–0.98) |
| All ideal  (vs. Any non-ideal) | - | 0.85 (0.71–1.02) |
| High CVH  (vs. Low CVH) | - | 0.79 (0.63–0.99) |
| Moderate CVH  (vs. Low CVH) | - | 0.88 (0.69–1.11) |
|  | - | Neonatal respiratory disease |
| CVH score | - | 0.94 (0.86–1.03) |
| All ideal  (vs. Any non-ideal) | - | 0.82 (0.65–1.04) |
| High CVH  (vs. Low CVH) | - | 0.73 (0.55–0.97) |
| Moderate CVH  (vs. Low CVH) | - | 0.80 (0.59–1.08) |
|  | - | Neonatal hypoglycemia |
| CVH score | - | 0.93 (0.80–1.09) |
| All ideal  (vs. Any non-ideal) | - | 0.86 (0.58–1.26) |
| High CVH  (vs. Low CVH) | - | 0.73 (0.46–1.17) |
| Moderate CVH  (vs. Low CVH) | - | 0.75 (0.45–1.24) |

^a^All analyses were adjusted for maternal age, gravidity, parity, occupation, income, education, and gestational week tested. ^b^Pregnant women with all ideal metrics. ^c^Pregnant women with one non-ideal metric. ^d^Pregnant women with at least one poor metric. ^e^Pregnant women with non-ideal metrics but no poor metrics.

**Supplementary Table 4. Participant characteristics according to data completeness**

|  | **Total**  (*N* = 2,775) | **Missing**  (*N* = 234) | ***P*** |
| --- | --- | --- | --- |
| Maternal age (years) | 30.88 ± 4.00 | 31.28 ± 4.01 | 0.146 |
| Gravidity | 1.83 ± 1.04 | 1.83 ± 1.00 | 0.990 |
| Parity | 0.32 ± 0.51 | 0.30 ± 0.51 | 0.632 |
| Occupation |  |  | 0.579 |
| Home | 282 (10.2) | 29 (12.4) |  |
| Low physical | 1,976 (71.2) | 163 (69.7) |  |
| Medium physical | 507 (18.3) | 42 (17.9) |  |
| High physical | 10 (0.4) | 0 (0.0) |  |
| Income |  |  | 0.017 |
| Low | 191 (6.9) | 10 (4.3) |  |
| Medium | 907 (32.7) | 61 (26.1) |  |
| High | 1,677 (60.4) | 163 (69.7) |  |
| Education |  |  | 0.246 |
| High school and below | 838 (30.2) | 61 (26.1) |  |
| Bachelor | 1,455 (52.4) | 124 (53.0) |  |
| Postgraduate | 482 (17.4) | 49 (20.9) |  |
| Gestational week tested | 25.27 ± 1.42 | 25.23 ± 2.60 | 0.751 |
| Birth gestational week | 38.84 ± 1.34 | 38.89 ± 1.39 | 0.573 |
| Birth weight (g) | 3,275.44 ± 430.82 | 3210.00 ± 428.00 | 0.026 |
| Apgar score 1 min | 9.82 ± 0.71 | 9.82 ± 0.57 | 0.946 |
| Apgar score 5 min | 9.96 ± 0.48 | 9.98 ± 0.13 | 0.442 |
| Newborn sex |  |  | 0.596 |
| Female | 1,307 (47.1) | 106 (45.3) |  |
| Male | 1,468 (52.9) | 128 (54.7) |  |
| Smoking grade |  |  | 0.611 |
| Poor | 11 (0.4) | 0 (0.0) |  |
| Intermediate | 28 (1.0) | 2 (0.9) |  |
| Ideal | 2,736 (98.6) | 232 (99.1) |  |
| Blood pressure grade |  |  | 0.397 |
| Poor | 32 (1.2) | 1 (0.8) |  |
| Intermediate | 670 (24.1) | 23 (19.0) |  |
| Ideal | 2,073 (74.7) | 97 (80.2) |  |
| Body mass index grade |  |  | 0.442 |
| Poor | 350 (12.6) | 18 (15.0) |  |
| Ideal | 2,425 (87.4) | 102 (85.0) |  |
| Fasting blood glucose grade |  |  | 0.150 |
| Poor | 121 (4.4) | 4 (2.2) |  |
| Ideal | 2,654 (95.6) | 181 (97.8) |  |
| Total cholesterol grade |  |  | 0.152 |
| Poor | 192 (6.9) | 16 (10.1) |  |
| Intermediate | 561 (20.2) | 37 (23.4) |  |
| Ideal | 2,022 (72.9) | 105 (66.5) |  |
| Sleep health grade |  |  | 0.370 |
| Poor | 198 (7.1) | 7 (6.4) |  |
| Intermediate | 486 (17.5) | 25 (22.7) |  |
| Ideal | 2,091 (75.4) | 78 (70.9) |  |

CVH, cardiovascular health.

Data are expressed as means ± standard deviations or numbers (percentages).

**Supplementary Table 5. Associations between gestational cardiovascular health and adverse maternal and/or neonatal outcomes** **using multiple imputation**

|  | **RR (95% CI)^a^** | **RR (95% CI)^a^** |
| --- | --- | --- |
|  | Maternal outcomes | Neonatal outcomes |
| CVH score | 0.93 (0.91–0.96) | 0.96 (0.93–0.98) |
| All ideal^b^  (vs. Any non-ideal^c^) | 0.86 (0.80–0.93) | 0.95 (0.87–1.03) |
| High CVH^b^  (vs. Low CVH^d^) | 0.80 (0.73–0.88) | 0.89 (0.81–0.98) |
| Moderate CVH^e^  (vs. Low CVH) | 0.89 (0.81–0.97) | 0.89 (0.81–0.97) |
|  | Hypertensive disorders of pregnancy | Large for gestational age |
| CVH score | 0.66 (0.62–0.70) | 0.85 (0.80–0.90) |
| All ideal  (vs. Any non-ideal) | 0.26 (0.17–0.39) | 0.88 (0.72–1.07) |
| High CVH  (vs. Low CVH) | 0.19 (0.12–0.29) | 0.67 (0.54–0.83) |
| Moderate CVH  (vs. Low CVH) | 0.51 (0.39–0.66) | 0.58 (0.46–0.73) |
|  | Intrahepatic cholestasis of pregnancy | Small for gestational age |
| CVH score | 0.90 (0.74–1.11) | 1.06 (0.95–1.19) |
| All ideal  (vs. Any non-ideal) | 0.88 (0.51–1.54) | 1.07 (0.79–1.45) |
| High CVH  (vs. Low CVH) | 0.99 (0.50–1.93) | 1.22 (0.84–1.77) |
| Moderate CVH  (vs. Low CVH) | 1.21 (0.63–2.31) | 1.23 (0.86–1.78) |
|  | Fetal distress | Macrosomia |
| CVH score | 1.05 (0.99–1.11) | 0.76 (0.67–0.85) |
| All ideal  (vs. Any non-ideal) | 1.10 (0.95–1.28) | 0.77 (0.52–1.12) |
| High CVH  (vs. Low CVH) | 1.10 (0.92–1.33) | 0.50 (0.33–0.76) |
| Moderate CVH  (vs. Low CVH) | 1.00 (0.83–1.21) | 0.39 (0.25–0.61) |
|  | Preterm birth | Low birth weight |
| CVH score | 0.92 (0.83–1.02) | 0.96 (0.84–1.10) |
| All ideal  (vs. Any non-ideal) | 0.90 (0.65–1.25) | 0.79 (0.48–1.29) |
| High CVH  (vs. Low CVH) | 0.82 (0.56–1.19) | 0.79 (0.45–1.38) |
| Moderate CVH  (vs. Low CVH) | 0.83 (0.57–1.21) | 1.01 (0.61–1.68) |
|  | Primary cesarean delivery | Admission to neonatal intensive care unit |
| CVH score | 0.93 (0.89–0.96) | 0.96 (0.92–0.99) |
| All ideal  (vs. Any non-ideal) | 0.83 (0.73–0.93) | 0.92 (0.83–1.02) |
| High CVH  (vs. Low CVH) | 0.77 (0.67–0.88) | 0.87 (0.77–0.98) |
| Moderate CVH  (vs. Low CVH) | 0.88 (0.77–1.00) | 0.90 (0.80–1.01) |
|  | - | Neonatal hyperbilirubinemia |
| CVH score | - | 0.92 (0.87–0.98) |
| All ideal  (vs. Any non-ideal) | - | 0.93 (0.77–1.12) |
| High CVH  (vs. Low CVH) | - | 0.83 (0.67–1.03) |
| Moderate CVH  (vs. Low CVH) | - | 0.81 (0.66–1.00) |
|  | - | Neonatal respiratory disease |
| CVH score | - | 0.93 (0.86–1.00) |
| All ideal  (vs. Any non-ideal) | - | 0.78 (0.61–1.00) |
| High CVH  (vs. Low CVH) | - | 0.70 (0.52–0.93) |
| Moderate CVH  (vs. Low CVH) | - | 0.82 (0.62–1.07) |
|  | - | Neonatal hypoglycemia |
| CVH score | - | 0.89 (0.78–1.01) |
| All ideal  (vs. Any non-ideal) | - | 0.68 (0.45–1.03) |
| High CVH  (vs. Low CVH) | - | 0.65 (0.40–1.07) |
| Moderate CVH  (vs. Low CVH) | - | 0.94 (0.61–1.45) |

^a^All analyses were adjusted for maternal age, gravidity, parity, occupation, income, education, and gestational week tested. ^b^Pregnant women with all ideal metrics. ^c^Pregnant women with one non-ideal metric. ^d^Pregnant women with at least one poor metric. ^e^Pregnant women with non-ideal metrics but no poor metrics.

**References**

38. Hedman, C., et al., *Effects of pregnancy on mothers' sleep.* Sleep Med, 2002. **3**: p. 37-42.

39. Izci-Balserak, B., et al., *Changes in Sleep Characteristics and Breathing Parameters During Sleep in Early and Late Pregnancy*. J Clin Sleep Med, 2018. **14**: p. 1161-1168.
